# Efficacy and safety of casirivimab-imdevimab combination on COVID-19 patients: A systematic review and meta-analysis randomized controlled trial

**DOI:** 10.1016/j.heliyon.2023.e22839

**Published:** 2023-11-27

**Authors:** Imam Adi Wicaksono, Cecep Suhandi, Khaled M. Elamin, Nasrul Wathoni

**Affiliations:** a Department of Pharmacology and Clinical Pharmacy, Universitas Padjadjaran, Sumedang 45363, West Java, Indonesia; b Department of Pharmaceutics and Pharmaceutical Technology, Universitas Padjadjaran, Sumedang 45363, West Java, Indonesia; c Graduate School of Pharmaceutical Sciences, Kumamoto University, Kumamoto 862-0973, Japan

**Keywords:** Casirivimab, Imdevimab, COVID-19, Efficacy, Safety

## Abstract

**Background:**

The advantages and disadvantages of casirivimab-imdevimab for coronavirus disease 2019 are not well understood. We conducted a systematic review and meta-analysis of relevant literature to determine the therapeutic effectiveness and potential side effects of casirivimab-imdevimab in COVID-19 patients.

**Methods:**

Databases were searched from the time of their commencement until February 28th, 2023. The primary results evaluated were the death rate at 28 days, progression of current clinical symptoms within 28 days, viral load, discharge from hospital, and any adverse events. Also, we contrasted the effects of the casirivimab-imdevimab treatment with placebo or standard of care. The protocol registration for this systematic review and meta-analysis was recorded in the PROSPERO database (CRD42023412835).

**Results:**

A total of eight studies were included, comprising 19,819 patients, and conducted a qualitative assessment of their risk of bias using the Cochrane risk of bias tool. Casirivimab-imdevimab effectively reduced the mortality rate (OR = 0.62; 95 % CI of 0.40–0.98; *p* = 0.04; I^2^ = 30 %) and reduced the progression of clinical symptoms (OR = 0.86; 95 % CI of 0.79–0.93; *p* = 0.0003; I^2^ = 57 %). Casirivimab-imdevimab also improved viral load clearance and hospital discharge. Additionally, the trials' findings demonstrated a slight decrease in the likelihood of adverse events occurring with the use of casirivimab-imdevimab.

**Conclusion:**

Our research suggests that casirivimab-imdevimab may be a valuable, safe, and effective anti-SARS-CoV-2 regimen.

## 1 Introduction

The investigation of therapies that can cure COVID-19 patients has progressed rapidly. Patients have a wide range of therapy choices available to them, including several regimens that can be used singly or in combination. Using antiviral medications to treat patients with COVID-19, especially those with severe symptoms, is recognized as being more effective than utilizing conventional medication [1,2]. Among these is molnupiravir, which can reduce the risk of all-cause death and enhance the proportion of patients with negative virus status in patients with mild or moderate severity [3,4]. Remdesivir, which operates by inhibiting RNA-dependent RNA polymerase (RdRp) using the same mechanism of action, is recognized as being beneficial in enhancing clinical condition, lowering mortality rates, and circumventing the need for high-flow supplemental oxygen and invasive mechanical ventilation in hospitalized patients [5,6]. Recently, the use of Paxlovid (consist of nilmatrelvir as main protease inhibitor and ritonavir as cytochrome P450 3A4 (CYP3A4) inhibitor) can also be an effective option in preventing death and improving the status of severe symptoms in elderly patients, immunocompromised patients, and patients with neurological and cardiovascular comorbidities [7,8]. However, the use of the available therapeutic options is also not without the risk of side effects, which are sometimes more severe than the effectiveness obtained.

Each of the available effective oral antiviral options is associated with varying safety concerns. Paxlovid usage is notably linked to a heightened incidence of skin toxicity [9]. Furthermore, extended utilization has been observed to correlate with embryonic toxicity and an elevated risk of life-threatening complications in pregnant patients [10]. Given its nature as a ribonucleoside analogue, molnupiravir is inseparable from the potential risk of inducing mutagenic effects on the host cell's DNA, thereby causing mutations [11]. Moreover, the utilization of molnupiravir is prominently associated with a substantial prevalence of approximately 50 % in causing diarrhea [12]. In a separate open-level study involving individuals with severe symptoms, serious adverse effects linked to the administration of remdesivir were observed. Several clinical studies have found that remdesivir was associated with a variety of side effects, including atrial fibrillation, hypotension, hypersensitivity responses, vomiting, nausea, elevated liver enzymes, haematological adverse effects, and metabolic side effects [13]. Thus, the lack of the available antiviral regimens demonstrate how crucial it is to develop effective and less toxic antiviral agents that effectively cure COVID-19 patients.

Currently, the emphasis on the advancement of SARS-CoV-2's antiviral has transitioned to monoclonal antibody preparations as an alternative approach to address the challenges associated with previously utilized therapies. Casirivimab and imdevimab are two human monoclonal antibodies that attach to distinct regions of the SARS-CoV-2 spike protein receptor-binding region, inhibiting the virus from invading host cells [14]. Casirivimab and imdevimab are two monoclonal antibodies of the human immunoglobulin G-1 (IgG1) class, which have not been altered in their Fc regions [15]. These two biomolecules have dissociation constants of 45.8 pM and 46.7 pM, respectively, and they bind to different parts of the SARS-CoV-2's receptor binding domain (RBD) on spike protein region. They prevent the attachment of the SARS-CoV-2 virus to host cells by blocking the receptor binding domain (RBD) of the spike protein from attaching to the ACE2 receptor.

The FDA has authorized the use of the combination of these two antibodies under emergency conditions to treat high risk COVID-19 patients which are developing severe symptoms, who exhibit mild to moderate symptoms [16]. According to an initial clinical trial, patients who received the antibody therapy had reduced viral load levels and experienced fewer hospitalizations and doctor visits [17]. However, the efficiency of casirivimab-imdevimab in treating COVID-19 patients is yet to be determined, and additional investigation is required to evaluate its clinical effectiveness and safety. Hence, this research aims to scrutinize randomized clinical trials that examine the capability of casirivimab-imdevimab in treating COVID-19 patients.

## 2 Materials and methods

### 2.1 Search strategy and selection criteria

Through the utilization of the specified search terms: (Covid OR SARS-CoV-2 OR “Coronavirus Disease”) AND (Casirivimab OR Imdevimab) AND “Randomized Controlled Trial”), we were able to locate all pertinent publications in PubMed, Scopus, and ScienceDirect. Databases up to February 28, 2023 were searched to find any pertinent RCTs assessing the efficacy and safety of the casirivimab-imdevimab combination in COVID-19 patients. Brief reports, letters, abstracts, observational studies, case reports, review articles, and opinion pieces were all excluded. All findings were imported into Microsoft Excel 2016 and duplicate articles were eliminated. Studies were limited to human as object study and published in English. The PRISMA (Preferred Reporting Items for Systematic Reviews and Meta-Analyses) statement was adhered to while carrying out this investigation (Table S1). The methodology for this systematic review and meta-analysis was recorded in the PROSPERO database with the identification number CRD42023412835.

Two reviewers (CS and IAW) with similar experience and skills looked at the titles and abstracts of the articles on their own. In cases where the information was unclear from the title and abstract, the entire document was retrieved. Any difference of opinion was discussed and settled by agreement with the third reviewer (NW). A study was deemed eligible if it fulfilled the following conditions: 1) It was a randomized controlled trial; 2) It presented information on significant outcomes in COVID-19 patients who were treated with casirivimab-imdevimab; and 3) It was compared to a placebo or standard of care.

The research in the appendices of this systematic review and meta-analysis was picked because it most likely fit the following PICO approach: Populations (P): COVID-19 patients; Interventions (I): treatment with casirivimab-imdevimab; Control (C): patients who only accept standard of care or a placebo; and Outcomes (O): primary outcomes: mortality at day 28, appearance of clinical symptoms at day 28, discharge alive at day 28, and least-squares mean change (log_10_ copies/mL) at day 7; secondary outcome: serious adverse events.

### 2.2 Data extraction and risk of bias analysis

Two reviewers (CS and IAW) collected data independently from selected papers. Extracted information included the following: 1) study characteristics, such as design, study locations, study dates, and primary outcome; 2) study population information, such as demographics of intervention and control arms group of patients; and 3) duration of follow-up and primary outcome. The suggested domains by the Cochrane collaboration were analyzed for potential risks of bias, specifically focusing on randomization, allocation concealment, outcomes assessment, blinding, and selective reporting [18,19]. Any discrepancies were settled through deliberation with a third reviewer (NW).

### 2.3 Data synthesis and statistical analysis

The statistical analysis followed the recommendations outlined in the PRISMA statement for systematic reviews and meta-analyses [20,21]. The Mantel-Haenszel technique was utilized to estimate the combined odds ratios (ORs) and their commensurate 95 % confidence intervals (CIs). To determine statistical significance, a threshold of a *p*-value ≤0.05 was set. The study used the Higgins and Thompson's I^2^ statistic to gauge heterogeneity, which reflects the degree of variation between trials attributed to differences among the trials themselves rather than sampling error. To determine the level of heterogeneity, I^2^ values were categorized as follows: 0–25 % for low, 26–75 % for moderate, and above 75 % for high levels of heterogeneity [22]. The Review Manager Version 5 was utilized for performing the statistical analysis. The random effect was utilized while heterogeneity of included studies was high and fixed effect was used while heterogeneity of included studies has a low-moderate level [22].

## 3 Results

### 3.1 Search results and characteristics of included studies

**Fig. 1:**
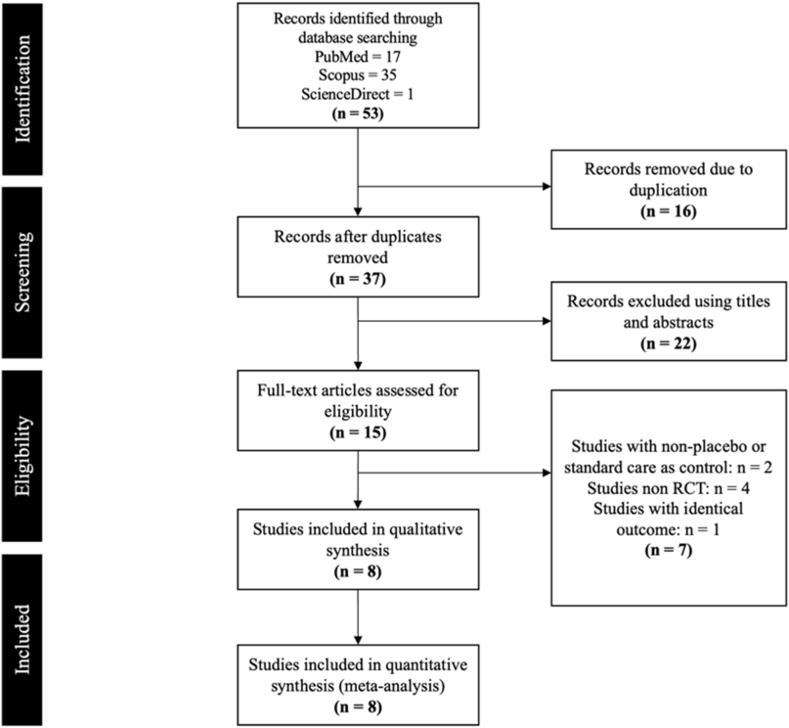
Visual representation of the study selection process based on PRISMA guideline.

The PRISMA flow chart, which summarizes the search approach, is shown in Fig. 1. After a comprehensive read, eight RCTs among the 15 full-text publications that were found through the literature search (Table S2 and Table S3) qualified for inclusion for further investigation [17,[23], [24], [25], [26], [27], [28], [29]]. The study included 19,819 patients, of whom 10,387 received casirivimab-imdevimab and 9432 received placebo or standard of care. The intervention and control groups shared similar baseline traits. Table 1 provides a detailed descriptions of included studies.

**Table 1:** Study characteristics.

Study	Site	Date	Design	Patient Characteristics			Dosage	Follow Up	Primary Outcome
				Patient Status	Setting	Vaccine Status			
Herman et al. [23]	USA, Romania, and Moldova	July 13, 2020, to Oct 4, 2021	Randomised, double-blind, placebo-controlled trial	Non-infected	Non-hospitalized	Non-vaccinated	CAS 600 mg + IMD 600 mg	228 days	Participants who were RT-PCR-negative
Horby & Landray [24]	UK	Sept 18, 2020, to May 22, 2021	Randomised, controlled, open-label platform trial	Infected	Hospitalized	Both vaccinated and non-vaccinated	CAS 4000 mg + IMD 4000 mg	28 days	Mortality at 28 days
Isa et al. [25]	USA	Cutoff date of May 21, 2021	Phase 1, double-blind, placebo-controlled study	Non-infected	Non-hospitalized	Non-vaccinated	CAS 600 mg + IMD 600 mg	28 weeks	The occurrence rate of adverse events (AEs) that are of special interest (AESIs), including severe injection site reactions (ISRs) or hypersensitivity reactions, during the first four days following the administration of CAS + IMD or a placebo, as well as the changes in the blood concentration of CAS + IMD over time.
O'Brien et al. [26]	USA, Romania, and Moldova	July 13, 2020, to January 28, 2021	Randomized, double-blind, placebo-controlled trial	Both infected and non-infected	Non-hospitalized	Non-vaccinated	CAS 600 mg + IMD 600 mg	28 days	The proportion of patients who tested negative for COVID-19 antibodies and subsequently developed symptoms of the disease.
Portal-Celhay et al. [27]	USA	December 15, 2020, to March 4, 2021	Randomized, double-blind, placebo-controlled, parallel-group, dose-ranging study	Infected	Non-hospitalized	Non-vaccinated	CAS 300 mg + IMD 300 mg and CAS 600 mg + IMD 600 mg	7 days	The average daily change in viral load (measured in log_10_ copies per milliliter) from the baseline to day 7, calculated as a time-weighted average.
Somersan-Karakaya et al. [28]	USA, Brazil, Chile, Mexico, Moldova, and Romania	June 10, 2020 to April 9, 2021	Adaptive, phase 1/2/3, double-blinded, placebo-controlled trial	Infected	Hospitalized	Both vaccinated and non-vaccinated	CAS 1200 mg + IMD 1200 mg and CAS 4000 mg + IMD 4000 mg	28 days	The endpoint for virologic efficacy was determined by calculating the time-weighted average of the daily change in viral load (measured from samples obtained from the nasopharynx) between day 1 and day 7 for the group of patients who tested negative for COVID-19 antibodies at baseline. The clinical efficacy endpoint refers to the percentage of patients who required mechanical ventilation or passed away during the period of days 6–29.
Weinreich et al. [17]	N/A	June 16, 2020, to August 13, 2020	Multicenter, randomized, double-blind, placebo-controlled, phase 1–3 clinical trial	Infected	Non-hospitalized	N/A	CAS 1200 mg + IMD 1200 mg and CAS 4000 mg + IMD 4000 mg	28 days	Virologic outcome: the average change in viral load from baseline to day 7, weighted by time (measured in log_10_ scale); Clinical outcome: the percentage of patients who made at least one visit to a healthcare facility.
Weinreich et al. [29]	N/A	September 24, 2020, to January 17, 2021	Phase 3 portion of multicenter, randomized, double-blind, placebo-controlled, phase 1–3 clinical trial	Infected	Non-hospitalized	N/A	CAS 600 mg + IMD 600 mg and CAS 1200 mg + IMD 1200 mg	28 days	Admission to the hospital or dying from any cause related to COVID-19.

All studies included in the analysis were conducted in multiple medical centers and involved patients diagnosed with COVID-19. The trials were carried out in various countries such as the USA, Romania, Moldova, the UK, Brazil, Chile, and Mexico. Among the studies analyzed, seven followed a double-blinded, placebo-controlled design, whereas one utilized an open-label platform approach [24]. Table 2 displays the particular study design and criteria.

**Table 2:** Study design and criteria.

Study	Group	Sample Size	Age (years)	Race/Ethnic group (%)			Sex (%)	
				White	Black or African American	Asian	Male	Female
Herman et al. [23]	CAS-IMD (1200 mg)	841	43.0 ± 26^a^	86.7	8.4	2.9	45.1	54.9
	Placebo	842	43.5 ± 24^a^	84.6	10.2	2.6	47.5	52.5
Horby & Landray [24]	CAS-IMD (8000 mg)	4839	61.9 ± 14.6^a^	78.0	N/A	N/A	63.0	37.0
	Standard of Care	4946	61.9 ± 14.4^a^	77.0	N/A	N/A	63.0	37.0
Isa et al. [25]	CAS-IMD (1200 mg)	729	48.0 (36.0–58.0)**	86.7	10.0	1.6	55.1	44.9
	Placebo	240	48.0 (36.0–59.0)**	86.3	10.0	2.1	55.0	45.0
O'Brien et al. [26]	CAS-IMD	753	43.2 (12–87)**	86.7	8.2	3.1	44.2	55.8
	Placebo	752	42.7 (12–92)**	84.4	10.4	2.5	47.6	52.4
Portal-Celhay et al. [27]	CAS + IMD (600 mg)	75	33.5 ± 10.88^a^	89.3	N/A	6.7	48.0	52.0
	CAS + IMD (1200 mg)	73	33.5 ± 9.18^a^	80.8	N/A	12.3	47.9	52.1
	Placebo	77	35.1 ± 9.97	83.1	N/A	13.0	40.3	59.7
Somersan-Karakaya et al. [28]	CAS + IMD (doses 2400 mg g and 8000 mg)	804	61.0 (20–98)**	63.4	12.3	3.9	54.4	45.6
	Placebo	393	64.0 (24–100)**	60.8	11.7	4.1	53.4	46.6
Weinreich et al. [17]	CAS + IMD (2400 mg)	92	43.0 (33.5–51.0) **	80.0	16.0	0.0	50.0	50.0
	CAS-IMD (8000 mg)	90	44.0 (36.0–53.0) **	87.0	7.0	1.0	42.0	58.0
	Placebo	93	45.0 (34.0–54.0)**	77.0	15.0	2.0	54.0	46.0
Weinreich et al. [29]	CAS + IMD (2400 g)	1355	50.0 (39.0–60.0)**	85.7	4.9	3.8	48.4	51.6
	CAS-IMD (1200 g)	736	48.5 (37.0–57.5)**	80.8	5.2	5.2	49.5	50.5
	Placebo 2400 g	1341	50.0 (37.0–58.0)**	84.7	4.9	4.2	47.2	52.8
	Placebo 1200 g	748	48.0 (35.0–57.0)**	81.7	5.1	4.8	47.1	52.9

a ) Mean ± SD; **) Median (IQR).

### 3.2 Risk of bias assessment

**Fig. 2:**
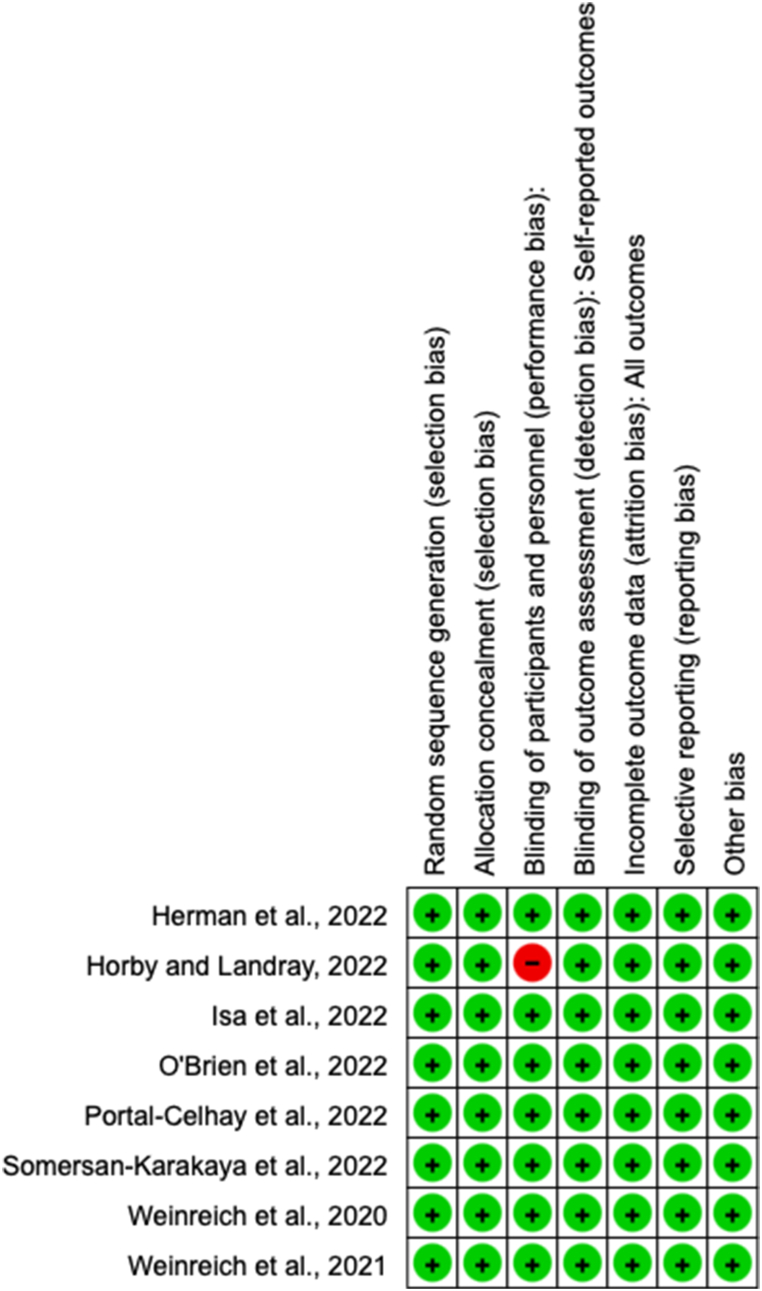
Risk of bias included studies.

Fig. 2 displays the assessment of the risk of bias for the trials that were included in the study. Allocation concealment and random sequence generation were both mentioned in all trials. An open-label research design was used in one investigation. The trial was at high risk of performance bias since both the participants and staff were aware of the treatment that was assigned [24]. Attrition bias risk was rated as being minimal across all experiments.

## 4 Assessment of primary outcomes

### 4.1 28-Day mortality

**Fig. 3:**
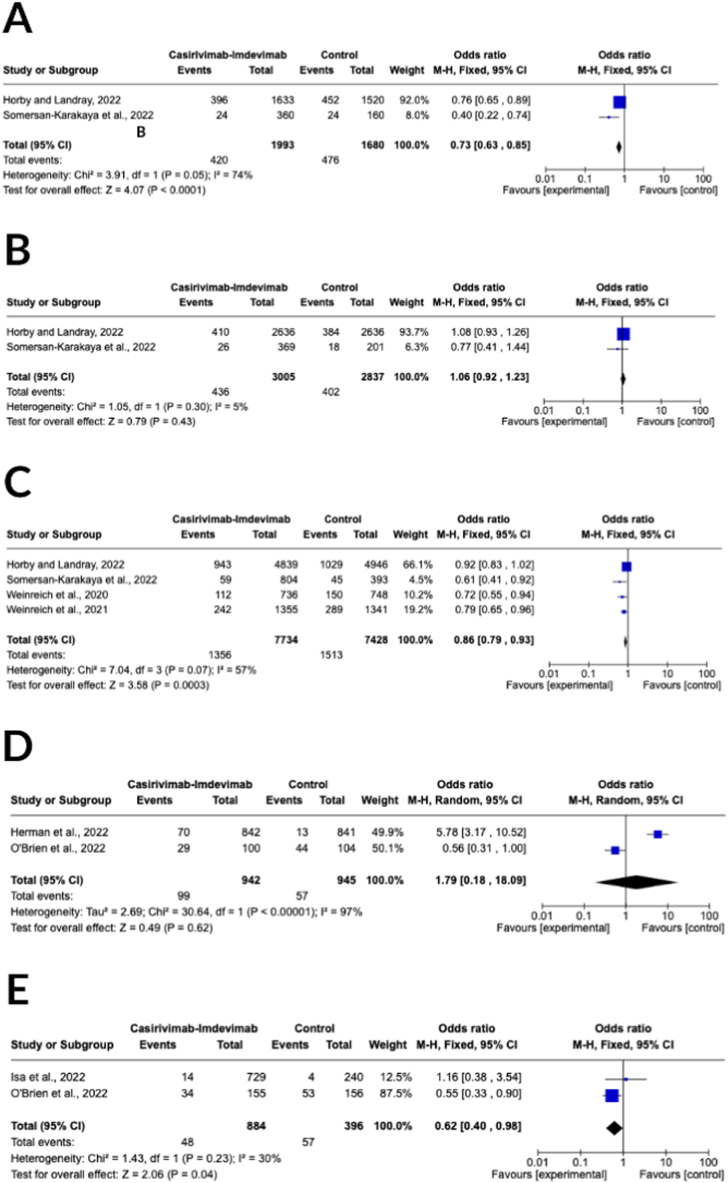
Forest plot for 28-day mortality outcome of **(A)** seronegative baseline, **(B)** seropositive baseline, and **(C)** overall baseline characteristics and progression to present clinical symptoms at 28-day of **(D)** seronegative baseline and **(E)** overall baseline characteristics.

Four RCTs investigated the 28-day mortality as an outcome measure [17,24,28,29]. The patients who were seronegative at the start of the study had a reduced mortality rate in the casirivimab-imdevimab cluster with 420 deaths out of 1993 participants (21.07 %), in contrast to the control group with 476 deaths out of 1680 participants (28.33 %), as demonstrated in Fig. 3A. In patients who were seropositive at the start of the study, the group that received casirivimab-imdevimab had a combined total of 436 deaths out of 3005 participants (14.51 %), while the control group had 402 deaths out of 2837 participants (14.17 %), as depicted in Fig. 3B. The combined analysis showed a statistically significant decrease in 28-day mortality with casirivimab-imdevimab treatment compared to control in patients with negative antibody status (odds ratio [OR] 0.73, 95 % confidence interval [CI] of 0.63–0.85; *p* < 0.0001), but not in those with positive antibody status (OR 1.06, 95 % CI 0.92–0.23; *p* = 0.43). However, after pooling all the baseline characteristics of the patients, a noteworthy reduction in 28-day mortality was observed in the casirivimab-imdevimab treatment group compared to the control group (OR 0.86, 95 % CI of 0.79–0.93; *p* = 0.0003) (shown in Fig. 3C). The casirivimab-imdevimab group had a total of 1356 deaths out of 7734 participants (17.53 %), while the control group had 1513 deaths out of 7428 participants (20.37 %). The heterogeneity was moderate for patients with seronegative status and all baseline characteristics analysis (I^2^ = 74 % and 57 %, respectively), and low for patients with positive antibody status analysis (I^2^ = 65 %).

### 4.2 Progression to present clinical symptoms at 28-day

Three randomized controlled trials (RCTs) provided data on progression to present clinical symptoms after 28 days [23,25,26]. In patients who had a negative baseline for antibodies, 10.51 % of the participants who received casirivimab-imdevimab therapy developed clinical symptoms at 28 days, which is higher than the 6.03 % of participants in the control group. The analysis did not show a significant difference between the two groups (OR 1.79, 95 % CI of 0.18–18.09; *p* = 0.62) (Fig. 3D). However, for patients with all baseline characteristics, 48 out of 884 participants (14.39 %) who received casirivimab-imdevimab therapy progressed to present clinical symptoms at 28 days compared to 57 out of 396 participants (9.01 %) in the control group, and this difference was statistically significant (OR 0.62, 95 % CI of 0.40–0.98; *p* = 0.04) (Fig. 3E). The analysis for patients with a seronegative baseline showed high heterogeneity (I^2^ = 97 %), whereas the analysis for patients with all baseline characteristics showed moderate heterogeneity (I^2^ = 30 %).

### 4.3 Viral load

**Fig. 4:**
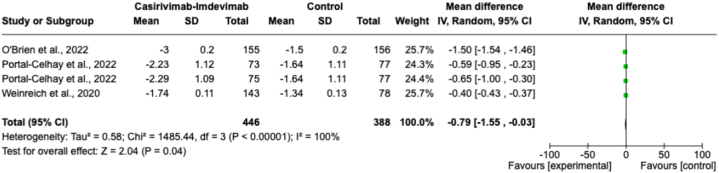
Forest plot for viral load at 7-day outcome.

The results of three randomized controlled trials (RCTs) on viral load, measured by least-squares mean change (log_10_ copies/ml), were analyzed [17,26,27]. A combined analysis showed that casirivimab-imdevimab therapy was more effective than the control in improving the outcome of viral load (mean difference of −0.79, 95 % CI -1.55, −0.03, *p* = 0.04) (see Fig. 4). However, the analysis showed high heterogeneity (I^2^ = 100 %) which means the studies were significantly different from each other.

### 4.4 28-Day discharge alive from hospital

**Fig. 5:**
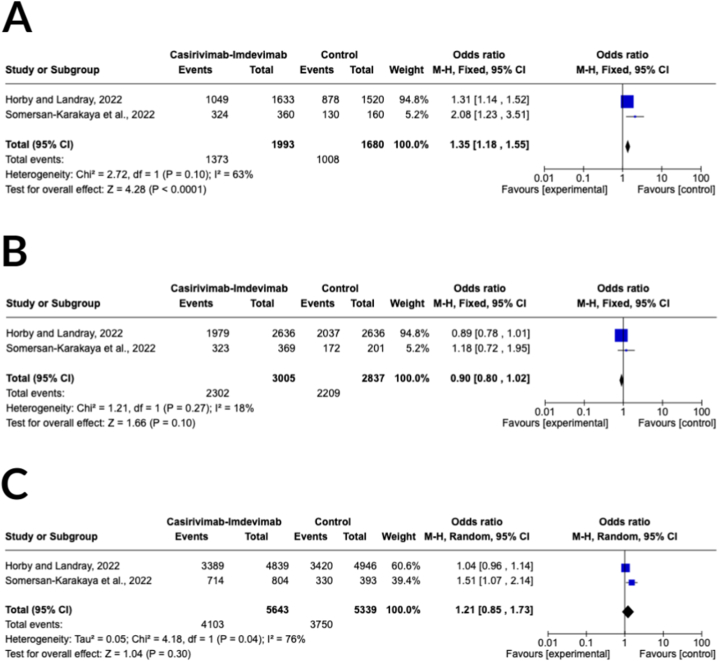
Forest plot for 28-day discharge alive from hospital outcome of **(A)** seronegative baseline, **(B)** seropositive baseline, and **(C)** overall baseline characteristics.

In two randomized controlled trials (RCTs), patients were assessed for being discharged alive from the hospital 28 days after hospital admission [24,28]. Furthermore, in patients with seropositive baseline characteristics, the casirivimab-imdevimab group had a total of 2302 events out of 3005 participants (76.61 %), while the control group had 2209 events out of 2837 participants (77.86 %). The combined analysis indicated a significant improvement in the likelihood of being discharged alive from the hospital within 28 days with casirivimab-imdevimab treatment compared to the control group in patients with seronegative baseline characteristics (OR 1.35, 95 % CI 1.18–1.55; *p* < 0.0001) as demonstrated in Fig. 5A, while no significant improvement was observed for seropositive baseline patients (OR 0.90, 95 % CI of 0.80–1.02; *p* = 0.10) as shown in Fig. 5B. However, pooled analysis for all baseline characteristics of patients showed a statistically insignificant improvement in 28-day discharge alive from hospital with casirivimab-imdevimab therapy than control (OR 1.21, 95 % CI of 0.85–1.73; *p* = 0.0003) (Fig. 5C). The casirivimab-imdevimab treatment arm had a total of 4103 events out of 5643 participants (72.71 %), while the control arm had 3750 events out of 5339 participants (70.24 %). Heterogeneity was considered moderate (I^2^ = 63 %), low (I^2^ = 18 %), and high (I^2^ = 76 %) in the analysis of patients with seronegative baseline, seropositive baseline, and all baseline characteristics, respectively.

## 5 Assessment of secondary outcomes

**Fig. 6:**
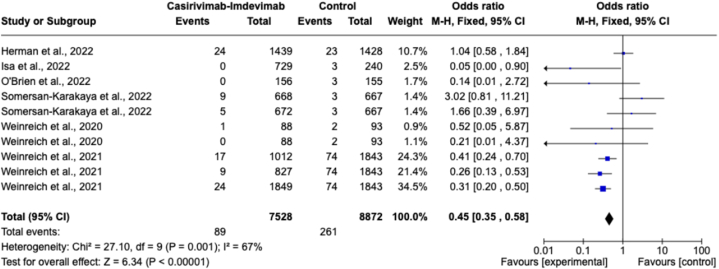
Forest plot for severe side effects.

Serious adverse events was assessed in this study as secondary outcomes to evaluate the safety aspect in the use of casirivimab-imdevimab. Six randomized controlled trials (RCTs) provided information on serious adverse events as a safety measure [17,23,25,26,28,29]. Out of a total of 7528 participants, 89 serious adverse events (68.89 %) were recorded in the casirivimab-imdevimab group, while 261 events (60.00 %) were reported in the control group consisting of 8872 participants. The odds of serious adverse events were slightly reduced in the casirivimab-imdevimab group compared to the control group (OR 0.45, 95 % CI 0.35–0.58; *p* < 0.00001). However, moderate heterogeneity was observed in the analysis (I^2^ = 67 %) as shown in Fig. 6.

## 6 Discussion

This study provided a detailed overview of the randomized trials that examined the safety and efficacy of casirivimab-imdevimab treatment for individuals with COVID-19. The findings of the study indicate a favourable trend towards reducing the mortality rates of COVID-19 patients treated with casirivimab-imdevimab therapy. Treatment with casirivimab-imdevimab was also linked to a decline in the course of COVID-19 clinical symptoms and viral load. The results consistently showed that among individuals with COVID-19, treatment with casirivimab-imdevimab was associated with increased rates of hospital discharge while alive and decreased likelihood of adverse events compared to the control group (which received either standard of care or a placebo). The findings of this meta-analysis study are consistent with prior research conducted by Hernandez et al., highlighting the efficacy of monoclonal antibodies as both a prophylactic agent and a therapeutic intervention [30]. It is noteworthy that the administration of monoclonal antibodies as a prophylactic agent demonstrates a reduction in viral load (denoted by a mean difference of −0.8 log_10_ copies). Furthermore, in the context of treatment, their utilization yields a substantial decrease in hospitalization rates among non-hospitalized patients (indicated by a risk ratio (RR) of 0.30), with the associated risk of serious adverse events being notably low (reflected in an RR of 0.47). Additionally, the present meta-analysis aligns with the findings from earlier meta-analysis studies conducted by Lin et al. [31]. This concordance underscores the consistent association between monoclonal antibody administration and a reduction in mortality rates (evidenced by an odds ratio (OR) of 0.16).

Casirivimab-imdevimab combination has been approved emergency use authorization for the treatment of COVID-19 by the FDA [32]. As reported by Deng et al., the use of casirivimab-imdevimab contributed to reduce mortality in seronegative baseline patients (OR 0.67, 95 % CI of 0.50; 0.91, *p*-value of 0.67) and hospitalization (OR 0.29, 95 % CI of 0.20; 0.42, *p-*value 0.65) [33]. It is well correlated with our findings that casirivimab-imdevimab given significant reduction in mortality at 28-day, both for seronegative baseline patients (27 %) and all baseline characteristics patients (14 %). Our findings also suggested that casirivimab-imdevimab well-correlated with improvement of hospitalized patients, as represented by 28-day discharge alive events, both for seronegative baseline patients (35 %) and all baseline characteristics patients (21 %). Our study also discovered an intriguing result that casirivimab-imdevimab therapy was linked to a lowered probability of serious unfavourable outcomes, which aligns with the outcomes of the research accomplished by Deng et al. [33]. The updates from this research are findings regarding defence against the progression of clinical symptoms of COVID-19 at 28 days and a significant reduction of the viral load profile compared to a control (a placebo or standard of care).

The IC_50_ value for casirivimab is 56.4 pM, while that of imdevimab is 165 pM [34]. The administration of these antibodies through subcutaneous injections leads to casirivimab having a half-life of 31.8 days, while imdevimab has a half-life of 26.9 days [35]. It supports our findings that casirivimab-imdevimab was a potential agent to provide protection and curing for COVID-19 patients, at least until 28 days after it was given. Casirivimab-imdevimab was also known to be effective as a prophylactic agent, especially for post-exposure patients [36]. The mechanism of action of this agent could be the best answer as to why it was effective to prevent the progression of COVID-19 infection. It could be due to the availability of casirivimab-imdevimab as human antibody substitutes before SARS-CoV-2 aggression.

Numerous investigations have demonstrated the utility of monoclonal antibodies as a therapeutic modality, particularly in the context of SARS-CoV-2, a virus characterized by a multitude of variants. Among these variants, the delta variant has garnered significant attention due to its notably heightened transmissibility and infection rates [37]. Furthermore, individuals afflicted with the delta variant have exhibited heightened disease severity in contrast to those with the omicron variant. While it is noteworthy that the present study did not conduct an analysis predicated on specific virus variants, previous research has substantiated the relative efficacy of monoclonal antibodies compared to conventional standard-of-care interventions [38]. Specifically, it has been established that casirivimab-imdevimab, while not surpassing sotrovimab in terms of efficacy in managing omicron variant infections, displays superior efficacy in both prophylactic and therapeutic contexts for patients afflicted with the delta variant, as evidenced by a risk ratio (RR) of 0.40 [38,39].

In terms of economic considerations, previous studies evaluating the utilization of casirivimab-imdevimab demonstrates a promising level of cost-effectiveness, particularly in the context of outpatient care [40]. In contrast to the conventional standard of medical care, the concomitant application of these two monoclonal antibodies yields substantial cost savings. Specifically, this approach leads to savings of €15.4 million attributed to a decrease in patient hospitalization rates, €59.3 million due to reduced utilization of intensive care services, and €20.3 million resulting from a decline in overall mortality rates [41]. Furthermore, the employment of preventive antibodies, a notable facet of casirivimab-imdevimab, contributes to diminished drug expenses by enhancing their efficacy. Through the administration of prophylactic monoclonal antibodies, potential reductions in treatment costs may amount to $275, with an associated efficacy rate exceeding 75 % [42]. It is worth noting, however, that comprehensive cost-effectiveness analyses comparing the use of casirivimab-imdevimab with alternative therapeutic strategies remain notably absent in the current literature.

Other than that, our meta-analysis has the following limitations: First, to verify our findings, more randomized, double-blind, multicenter trials are needed. Second, compared to other RCTs, the RECOVERY trial had a significantly larger patient enrolment [26]. Third, there were variations in the selection criteria, clinical practice variability across various geographic regions, and clinical progress measurement. Significant heterogeneity was also present in the statistical analyses. Fourth, because participants and staff were not blinded to the intervention in one of the included trials, there was a substantial risk of performance bias [24]. Fifth, different research uses different methods to determine the severity of patients at baseline. Therefore, further research employing consistent severity evaluation techniques is still needed. Finally, the majority of recent investigations on the safety of casirivimab-imdevimab were conducted over a short period of time. Therefore, further investigation is required to establish how common long-term negative reactions are.

## 7 Conclusion

The use of casirivimab-imdevimab shows promise as a safe treatment for SARS-CoV-2. However, additional research is necessary to investigate its therapeutic potential in patients with varying levels of severity and against emerging variants. As more research is conducted, it is possible that casirivimab-imdevimab will become more widely available in the future. Additionally, platform trials that compare casirivimab-imdevimab with other antibody regimens could be helpful in treating COVID-19.

## Declarations

### Funding

None.

## Consent for publication

Not applicable.

## Ethics approval and consent to participate

Ethical approval was not needed because this is a meta-analysis.

## Availability of data and material

All of relatable data can be requested upon reasonable request from the corresponding author.

## CRediT authorship contribution statement

**Imam Adi Wicaksono:** Conceptualization, Formal analysis, Validation, Writing – review & editing, Funding acquisition, Project administration. **Cecep Suhandi:** Conceptualization, Formal analysis, Methodology, Software, Writing – original draft. **Khaled M. Elamin:** Investigation, Validation, Writing – review & editing. **Nasrul Wathoni:** Formal analysis, Investigation, Validation, Writing – review & editing.

## Declaration of competing interest

The authors declare that they have no known competing financial interests or personal relationships that could have appeared to influence the work reported in this paper.
