# Supplementary material for: Efficacy and safety of casirivimab-imdevimab combination on COVID-19 patients: A systematic review and meta-analysis randomized controlled trial
Source: Heliyon. 2023 Nov 27;9(12):e22839. doi: 10.1016/j.heliyon.2023.e22839 (PMC10696184; doi:10.1016/j.heliyon.2023.e22839)
Supplement: Multimedia component 1 [file mmc1.docx]

**Table S1.** PRISMA Checklist

| **Section and Topic** | **Item #** | **Checklist item** | **Location where item is reported** |
| --- | --- | --- | --- |
| **TITLE** | | |  |
| Title | 1 | Identify the report as a systematic review. | Page 1 Line 3-4 |
| **ABSTRACT** | | |  |
| Abstract | 2 | See the PRISMA 2020 for Abstracts checklist. | **✓** |
| **INTRODUCTION** | | |  |
| Rationale | 3 | Describe the rationale for the review in the context of existing knowledge. | Page 4 Line 65-77 |
| Objectives | 4 | Provide an explicit statement of the objective(s) or question(s) the review addresses. | Page 4 Lines 76-77 |
| **METHODS** | | |  |
| Eligibility criteria | 5 | Specify the inclusion and exclusion criteria for the review and how studies were grouped for the syntheses. | Page 4-5, Lines 84-95 |
| Information sources | 6 | Specify all databases, registers, websites, organisations, reference lists and other sources searched or consulted to identify studies. Specify the date when each source was last searched or consulted. | Page 4, Lines 81-86 |
| Search strategy | 7 | Present the full search strategies for all databases, registers and websites, including any filters and limits used. | Supplemental file, Table S2 |
| Selection process | 8 | Specify the methods used to decide whether a study met the inclusion criteria of the review, including how many reviewers screened each record and each report retrieved, whether they worked independently, and if applicable, details of automation tools used in the process. | Page 5, Lines 93-96 |
| Data collection process | 9 | Specify the methods used to collect data from reports, including how many reviewers collected data from each report, whether they worked independently, any processes for obtaining or confirming data from study investigators, and if applicable, details of automation tools used in the process. | Page 5, Line 107 |
| Data items | 10a | List and define all outcomes for which data were sought. Specify whether all results that were compatible with each outcome domain in each study were sought (e.g. for all measures, time points, analyses), and if not, the methods used to decide which results to collect. | Page 5, Lines 103-105 |
|  | 10b | List and define all other variables for which data were sought (e.g. participant and intervention characteristics, funding sources). Describe any assumptions made about any missing or unclear information. | Page 5, Lines 107 – 111 |
| Study risk of bias assessment | 11 | Specify the methods used to assess risk of bias in the included studies, including details of the tool(s) used, how many reviewers assessed each study and whether they worked independently, and if applicable, details of automation tools used in the process. | Page 5, Lines 111 – 114 |
| Effect measures | 12 | Specify for each outcome the effect measure(s) (e.g. risk ratio, mean difference) used in the synthesis or presentation of results. | Page 6, Lines 117 – 119 |
| Synthesis methods | 13a | Describe the processes used to decide which studies were eligible for each synthesis (e.g. tabulating the study intervention characteristics and comparing against the planned groups for each synthesis (item #5)). | Page 4-5, Lines 84-95 |
|  | 13b | Describe any methods required to prepare the data for presentation or synthesis, such as handling of missing summary statistics, or data conversions. | Page 6, Lines 117 – 123 |
|  | 13c | Describe any methods used to tabulate or visually display results of individual studies and syntheses. | Page 6, Lines 119 – 122 |
|  | 13d | Describe any methods used to synthesize results and provide a rationale for the choice(s). If meta-analysis was performed, describe the model(s), method(s) to identify the presence and extent of statistical heterogeneity, and software package(s) used. | Page 6, Lines 117 – 126 |
|  | 13e | Describe any methods used to explore possible causes of heterogeneity among study results (e.g. subgroup analysis, meta-regression). | Page 6, Lines 119 – 123 |
|  | 13f | Describe any sensitivity analyses conducted to assess robustness of the synthesized results. | N/A |
| Reporting bias assessment | 14 | Describe any methods used to assess risk of bias due to missing results in a synthesis (arising from reporting biases). | Page 5-6, Lines 111-114 |
| Certainty assessment | 15 | Describe any methods used to assess certainty (or confidence) in the body of evidence for an outcome. | Page 6, Lines 117 – 119 |
| **RESULTS** | | |  |
| Study selection | 16a | Describe the results of the search and selection process, from the number of records identified in the search to the number of studies included in the review, ideally using a flow diagram. | Figure 1. |
|  | 16b | Cite studies that might appear to meet the inclusion criteria, but which were excluded, and explain why they were excluded. | Supplemental file, Table S3 |
| Study characteristics | 17 | Cite each included study and present its characteristics. | Table 1 |
| Risk of bias in studies | 18 | Present assessments of risk of bias for each included study. | Figure 2 |
| Results of individual studies | 19 | For all outcomes, present, for each study: (a) summary statistics for each group (where appropriate) and (b) an effect estimate and its precision (e.g. confidence/credible interval), ideally using structured tables or plots. | Figure 3-6 |
| Results of syntheses | 20a | For each synthesis, briefly summarise the characteristics and risk of bias among contributing studies. | Figure 2 |
|  | 20b | Present results of all statistical syntheses conducted. If meta-analysis was done, present for each the summary estimate and its precision (e.g. confidence/credible interval) and measures of statistical heterogeneity. If comparing groups, describe the direction of the effect. | Figure 3-6 |
|  | 20c | Present results of all investigations of possible causes of heterogeneity among study results. | Figure 3-6 |
|  | 20d | Present results of all sensitivity analyses conducted to assess the robustness of the synthesized results. | N/A |
| Reporting biases | 21 | Present assessments of risk of bias due to missing results (arising from reporting biases) for each synthesis assessed. | N/A |
| Certainty of evidence | 22 | Present assessments of certainty (or confidence) in the body of evidence for each outcome assessed. | N/A |
| **DISCUSSION** | | |  |
| Discussion | 23a | Provide a general interpretation of the results in the context of other evidence. | Page 9, Lines 217 – 225 |
|  | 23b | Discuss any limitations of the evidence included in the review. | Page 10, Lines 257-258 |
|  | 23c | Discuss any limitations of the review processes used. | Page 10, Lines 256-267 |
|  | 23d | Discuss implications of the results for practice, policy, and future research. | Page 10, Lines 270-275 |
| **OTHER INFORMATION** | | |  |
| Registration and protocol | 24a | Provide registration information for the review, including register name and registration number, or state that the review was not registered. | Page 5, Lines 90-92 |
|  | 24b | Indicate where the review protocol can be accessed, or state that a protocol was not prepared. | Page 5, Lines 103-105. |
|  | 24c | Describe and explain any amendments to information provided at registration or in the protocol. | N/A |
| Support | 25 | Describe sources of financial or non-financial support for the review, and the role of the funders or sponsors in the review. | Page 11, Line 278-279 |
| Competing interests | 26 | Declare any competing interests of review authors. | Page 11, Line 284-285 |
| Availability of data, code and other materials | 27 | Report which of the following are publicly available and where they can be found: template data collection forms; data extracted from included studies; data used for all analyses; analytic code; any other materials used in the review. | Page 11, Line 288-289 |

*From:*  Page MJ, McKenzie JE, Bossuyt PM, Boutron I, Hoffmann TC, Mulrow CD, et al. The PRISMA 2020 statement: an updated guideline for reporting systematic reviews. BMJ 2021;372:n71. doi: 10.1136/bmj.n71

For more information, visit: <http://www.prisma-statement.org/>

**Table S2.** Search Term Results

| String | Search Terms | Hits |
| --- | --- | --- |
| PubMed | (covid OR sars-cov-2 OR "coronavirus disease") AND (casirivimab OR imdevimab) [Title/Abstract/Keywords] limited to Randomized Controlled Trial | 17 |
| Scopus | (covid OR sars-cov-2 OR "coronavirus disease") AND (casirivimab OR imdevimab) AND "randomized controlled trial") [Title/Abstract/Keywords] | 35 |
| ScienceDirect | (covid OR sars-cov-2 OR "coronavirus disease") AND (casirivimab OR imdevimab) AND "randomized controlled trial") [Title/Abstract/Keywords] | 1 |

**Table S3.** Excluded articles.

| **Author** | **Title** | **Year** | **Reason for Exclusion** |
| --- | --- | --- | --- |
| Huang DT, McCreary EK, Bariola JR, Minnier TE, Wadas RJ, Shovel JA, Albin D, Marroquin OC, Kip KE, Collins K, Schmidhofer M, Wisniewski MK, Nace DA, Sullivan C, Axe M, Meyers R, Weissman A, Garrard W, Peck-Palmer OM, Wells A, Bart RD, Yang A, Berry LR, Berry S, Crawford AM, McGlothlin A, Khadem T, Linstrum K, Montgomery SK, Ricketts D, Kennedy JN, Pidro CJ, Nakayama A, Zapf RL, Kip PL, Haidar G, Snyder GM, McVerry BJ, Yealy DM, Angus DC, Seymour CW | Effectiveness of Casirivimab-Imdevimab and Sotrovimab During a SARS-CoV-2 Delta Variant Surge: A Cohort Study and Randomized Comparative Effectiveness Trial | 2022 | A study design was not RCT and using other antibody as comparator (neither placebo or standard of care) |
| Flaxman AD, Issema R, Barnabas RV, Ross JM | Estimated Health Outcomes and Costs of COVID-19 Prophylaxis With Monoclonal Antibodies Among Unvaccinated Household Contacts in the US | 2022 | Study was an analytical decision of RCT’s database |
| Mazzaferri F, Mirandola M, Savoldi A, De Nardo P, Morra M, Tebon M, Armellini M, De Luca G, Calandrino L, Sasset L, D'Elia D, Sozio E, Danese E, Gibellini D, Monne I, Scroccaro G, Magrini N, Cattelan A, Tascini C; MANTICO Working Group; Tacconelli E | Exploratory data on the clinical efficacy of monoclonal antibodies against SARS-CoV-2 Omicron variant of concern | 2022 | Study using other antibody as comparator (neither placebo or standard of care) |
| Balasubramanian P, Berto CG, Hassan E, Valentine N, De Vos G, Coyle C, Sydney E | Outcomes in Patients with Mild COVID-19 Treated With Casirivimab and Imdevimab or Bamlanivimab - A Single-Center Retrospective Cohort Study in the Bronx | 2022 | A study design was not RCT, but a retrospective cohort study |
| O'Brien MP, Forleo-Neto E, Musser BJ, Isa F, Chan KC, Sarkar N, Bar KJ, Barnabas RV, Barouch DH, Cohen MS, Hurt CB, Burwen DR, Marovich MA, Hou P, Heirman I, Davis JD, Turner KC, Ramesh D, Mahmood A, Hooper AT, Hamilton JD, Kim Y, Purcell LA, Baum A, Kyratsous CA, Krainson J, Perez-Perez R, Mohseni R, Kowal B, DiCioccio AT, Stahl N, Lipsich L, Braunstein N, Herman G, Yancopoulos GD, Weinreich DM; Covid-19 Phase 3 Prevention Trial Team | Subcutaneous REGEN-COV antibody combination to prevent Covid-19 | 2021 | Study has similar outcome with late phase trial (O’Brien, 2022) |
| McCreary EK, Bariola JR, Minnier TE, Wadas RJ, Shovel JA, Albin D, Marroquin OC, Kip KE, Collins K, Schmidhofer M, Wisniewski MK, Nace DA, Sullivan C, Axe M, Meyers R, Weissman A, Garrard W, Peck-Palmer OM, Wells A, Bart RD, Yang A, Berry LR, Berry S, Crawford AM, McGlothlin A, Khadem T, Linstrum K, Montgomery SK, Ricketts D, Kennedy JN, Pidro CJ, Haidar G, Snyder GM, McVerry BJ, Yealy DM, Angus DC, Nakayama A, Zapf RL, Kip PL, Seymour CW, Huang DT | The comparative effectiveness of COVID-19 monoclonal antibodies: A learning health system randomized clinical trial | 2022 | A study was a platform trial of RCT |
| Copin R, Baum A, Wloga E, Pascal KE, Giordano S, Fulton BO, Zhou A, Negron N, Lanza K, Chan N, Coppola A, Chiu J, Ni M, Wei Y, Atwal GS, Hernandez AR, Saotome K, Zhou Y, Franklin MC, Hooper AT, McCarthy S, Hamon S, Hamilton JD, Staples HM, Alfson K, Carrion R Jr, Ali S, Norton T, Somersan-Karakaya S, Sivapalasingam S, Herman GA, Weinreich DM, Lipsich L, Stahl N, Murphy AJ, Yancopoulos GD, Kyratsous CA | The monoclonal antibody combination REGEN-COV protects against SARS-CoV-2 mutational escape in preclinical and human studies | 2021 | Study using animal and human as subject of study. Evaluation using human as subject of study was not RCT |
